# Chemoperception of Specific Amino Acids Controls Phytopathogenicity in Pseudomonas syringae pv. tomato

**DOI:** 10.1128/mBio.01868-19

**Published:** 2019-10-01

**Authors:** Jean Paul Cerna-Vargas, Saray Santamaría-Hernando, Miguel A. Matilla, José Juan Rodríguez-Herva, Abdelali Daddaoua, Pablo Rodríguez-Palenzuela, Tino Krell, Emilia López-Solanilla

**Affiliations:** a Centro de Biotecnología y Genómica de Plantas, Universidad Politécnica de Madrid-Instituto Nacional de Investigación y Tecnología Agraria y Alimentaria, Pozuelo de Alarcón, Madrid, Spain; b Departamento de Biotecnología-Biología Vegetal, Escuela Técnica Superior de Ingeniería Agronómica, Alimentaria y de Biosistemas, Universidad Politécnica de Madrid, Madrid, Spain; c Department of Environmental Protection, Estación Experimental del Zaidín, Consejo Superior de Investigaciones Científicas, Granada, Spain; Centre National de la Recherche Scientifique; Institut Pasteur

**Keywords:** chemoreceptors, *Pseudomonas syringae*, virulence

## Abstract

Chemotaxis has been associated with the pathogenicity of bacteria in plants and was found to facilitate bacterial entry through stomata and wounds. However, knowledge regarding the plant signals involved in this process is scarce. We have addressed this issue using Pseudomonas syringae pv. tomato, which is a foliar pathogen that causes bacterial speck in tomato. We show that the chemoreceptor P. syringae pv. tomato PscA (PsPto-PscA) recognizes specifically and with high affinity l-Asp, l-Glu, and d-Asp. The mutation of the chemoreceptor gene largely reduced chemotaxis to these ligands but also altered cyclic di-GMP (c-di-GMP) levels, biofilm formation, and motility, pointing to cross talk between different chemosensory pathways. Furthermore, the PsPto-PscA mutant strain showed reduced virulence in tomato. Asp and Glu are the most abundant amino acids in plants and in particular in tomato apoplasts, and we hypothesize that this receptor may have evolved to specifically recognize these compounds to facilitate bacterial entry into the plant. Infection assays with the wild-type strain showed that the presence of saturating concentrations of d-Asp also reduced bacterial virulence.

## Abstract (precis)

There is substantive evidence that chemotaxis is a key requisite for efficient pathogenesis in plant pathogens. However, information regarding particular bacterial chemoreceptors and the specific plant signal that they sense is scarce. Our work shows that the phytopathogenic bacterium Pseudomonas syringae pv. tomato mediates not only chemotaxis but also the control of pathogenicity through the perception of the plant abundant amino acids Asp and Glu. We describe the specificity of the perception of l- and d-Asp and l-Glu by the PsPto-PscA chemoreceptor and the involvement of this perception in the regulation of pathogenicity-related traits. Moreover, a saturating concentration of d-Asp reduces bacterial virulence, and we therefore propose that ligand-mediated interference of key chemoreceptors may be an alternative strategy to control virulence.

## INTRODUCTION

Chemosensory pathways are widely distributed among bacteria and exert a key role in signal transduction processes associated with the response to environmental cues (1–3). The core of a chemosensory pathway is formed by a complex composed of a chemoreceptor, or methyl-accepting chemotaxis protein (MCP), the CheA histidine kinase, and the CheW adaptor protein. In the canonical pathway, signal binding to the chemoreceptor ligand binding domain (LBD) creates a molecular stimulus that modulates CheA autophosphorylation activity, which in turn alters the transphosphorylation activity of the response regulator CheY. In the case of a chemotaxis pathway, phosphorylated CheY (CheY-P) binds to the flagellar motor, causing ultimately chemotaxis (4–7). Although most chemosensory pathways appear to be involved in chemotaxis (8), other pathways were found to be associated with type IV pilus-based motility or the control of cyclic di-GMP (c-di-GMP) and cAMP second messenger levels (9–12).

Typically, the specificity of a chemotactic response is determined by signal recognition at the chemoreceptor LBD. Although chemoreceptors employ more than 80 different LBD types, at the structural level, they fall into two major families, namely, domains with parallel helix architecture (4HB, HBM, NIT, PilJ, and CHASE3) or domains with a central curved β-sheet (sCACHE, dCACHE, PAS, and GAF) (3). First relationships between LBD types and the nature of their cognate ligands are emerging (2, 3, 13).

The presence of chemosensory signaling genes in a bacterium depends on bacterial lifestyle (14, 15). Overall, approximately one-half of all bacteria were found to harbor chemosensory signaling genes (8). These species possess on average 14 chemoreceptor genes (8, 15). Similar estimates on chemoreceptor gene numbers have been obtained for human and animal pathogens (16). However, in marked contrast are plant pathogens, of which approximately 90% possess chemosensory signaling genes and on average 33 chemoreceptor genes, which is well superior to the bacterial average (16). The abundance of chemosensory signaling genes in phytopathogenic bacteria is consistent with the observation that the inactivation of chemotactic signaling causes in most cases a drop in virulence (17–20). It was concluded that chemotaxis is an important trait in early stages of infection, enabling bacterial entry into plants through natural openings like stomata or through wounds (16).

Despite their abundance and importance in the infection process, there is a paucity of information on the signals recognized by phytopathogen chemoreceptors. In Dickeya dadantii 3937, chemoattraction and chemorepellence to sugars, amino acids, and plant hormones like jasmonic acid have been associated with virulence (21). In Ralstonia solanacearum, chemotaxis to malate and aerotaxis were identified as being necessary for optimal virulence (19, 22). Furthermore, chemotactic behavior toward several compounds has been described for Pseudomonas syringae (23, 24) and Xanthomonas campestris (25). However, the role of particular chemoreceptors in the interaction with the host has so far been little investigated.

We have addressed this issue here using P. syringae pv. tomato DC3000 as a model. This strain has 49 chemoreceptors and four copies of the core chemosensory signaling proteins, suggesting the existence of 4 chemosensory pathways (26).

P. syringae pv. tomato is the causal agent of bacterial speck in tomato (27, 28). The main virulence determinant in this bacterium is the type III secretion system (T3SS) and the type III effector proteins (T3Es) (27). P. syringae pv. tomato is a saprophyte found in plant debris, soil, and leaf surfaces but is a weak epiphyte compared with other P. syringae strains (29). Therefore, mechanisms of adaptation and response to favorable conditions are central to ensure bacterial entry into the plant and concomitantly for disease development (30). Knowledge of these mechanisms is scarce, although it is known that motility contributes to bacterial entry through stomata during the first stages of the infection (31, 32). Biofilm formation is also associated with bacterial adaptation to environmental stress such as that generated by the interaction of P. syringae with plants (33). As in other bacteria, motility and biofilm formation are inversely regulated in P. syringae pv. tomato (34, 35), and pathogenicity was found to be associated with increased motility and a low degree of cell aggregation (36).

**FIG 1:**
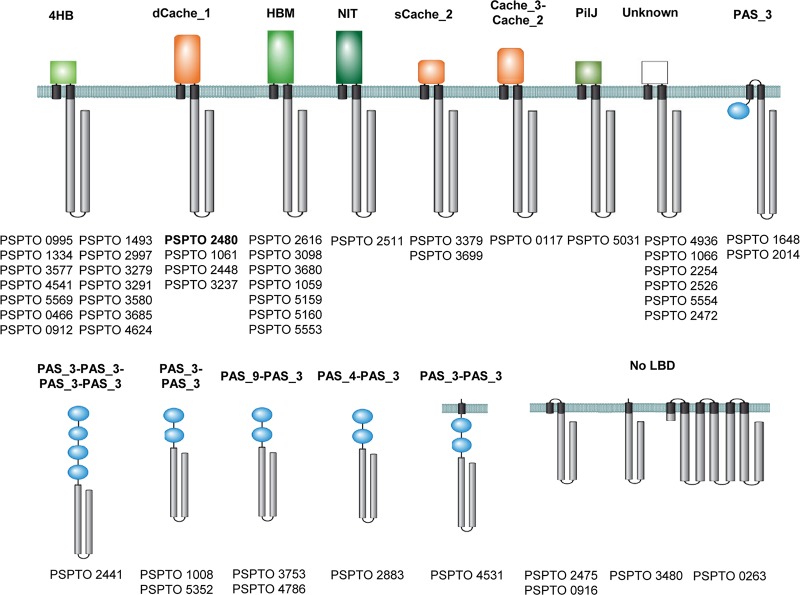
Chemoreceptor repertoire of P. syringae pv. tomato. LBDs were annotated according to the Pfam database (https://pfam.xfam.org/). The receptor studied is highlighted in boldface type.

Out of 49 P. syringae pv. tomato chemoreceptors, 36 showed the canonical topology, with a periplasmic LBD flanked by two transmembrane (TM) regions, typical for sensing extracytoplasmic signals. Among them, the very large majority of chemoreceptor LBDs are of the parallel helix type (4HB, HBM, Nit, and PilJ), and only a few form the curved β-sheet (sCACHE and dCACHE) (Fig. 1).

Nine chemoreceptors possess one or multiple PAS domains of cytosolic location that are likely to be involved in the sensing of cytosolic signals, such as the redox state or oxygen (37). Four other chemoreceptors are membrane bound but lack LBDs, and it is hypothesized that they respond to physicochemical stimuli like temperature or osmotic pressure. None of the P. syringae pv. tomato chemoreceptors have been characterized. However, the P. syringae pv. tomato chemoreceptors PSPTO_2480, PSPTO_1061, and PSPTO_2448 (Fig. 1) are homologous to the amino acid receptors PscA, PscB, and PscC of P. syringae pv. actinidiae (38) and PctA, PctB, and PctC of Pseudomonas aeruginosa (39–41). dCACHE LBD-containing amino acid chemoreceptors show a wide phylogenetic distribution and were identified, for example, in Halobacterium salinarum (42), Bacillus subtilis (43), Vibrio cholerae (44), Sinorhizobium meliloti (45), and Campylobacter jejuni (46), pointing to an important biological role of this chemoreceptor type (47).

In this work, we have determined the ligand profile of P. syringae pv. tomato PscA (PsPto-PscA) and showed that it exerts a double function, namely, in mediating chemotaxis and in modulating c-di-GMP levels, causing alterations in biofilm development. This receptor was found to play a key role in the infection process, and receptor saturation with its cognate ligands is proposed as an alternative strategy to control virulence.

## RESULTS

### PsPto-PscA binds l-Asp, d-Asp, and l-Glu.

**FIG 2:**
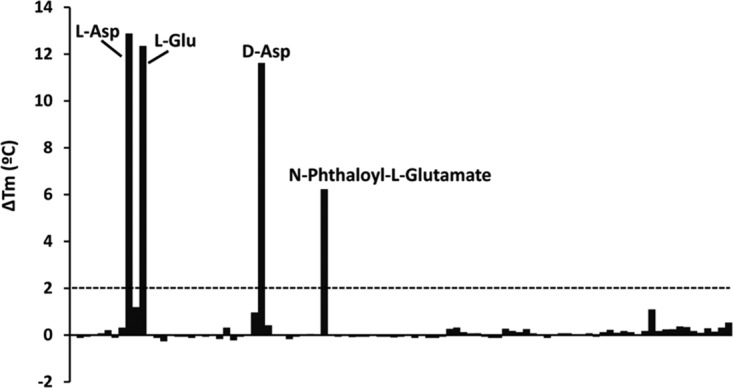
Differential scanning fluorimetry-based ligand screening of PsPto-PscA-LBD. Shown are the melting temperature (*T_m_*) changes for each of the 95 compounds present in the Biolog PM3B compound array of nitrogen sources with respect to the *T_m_* of the ligand-free protein. The dashed line indicates the threshold of 2°C for significant hits. Data are the means and standard deviations from two assays.

To identify the ligands recognized by PsPto-PscA, the individual LBD was expressed in Escherichia coli and purified from the soluble protein fraction using affinity chromatography. PsPto-PscA-LBD was then submitted to thermal shift assays in which alterations in thermal stability following ligand binding are monitored (48). The method permits the determination of the *T_m_* (melting temperature) value, and ligand-induced *T_m_* increases of more than 2°C are considered significant. The *T_m_* of the ligand-free protein was 37.6°C, and significant *T_m_* increases were observed for l-Asp, d-Asp, l-Glu, and *N*-phthaloyl-l-glutamate, while no *T_m_* increase was observed for any of the other proteinogenic amino acids or for d-Ala, d-Asn, d-Glu, d-Lys, d-Ser, or d-Val (Table 1 and Fig. 2).

**TABLE 1:** Δ*T_m_* values obtained by differential scanning fluorimetry and thermodynamic parameters for titration of PsPto-PscA-LBD with acid amino acids and their amides^*a*^

Ligand	Δ*T_m_* (°C)	Mean *K_D_* (μM) ± SEM	Mean Δ*H* (kcal/mol)± SEM
**l-Asp**	+12.88	1.2 ± 0.1	−6.7 ± 0.1
**d-Asp**	+11.63	1.2 ± 0.1	−4.7 ± 0.1
l-Asn	+0.31	No binding	
d-Asn	+0.97	No binding	
**l-Glu**	+12.35	3.4 ± 0.1	−7.8 ± 0.1
d-Glu	+0.42	No binding	
l-Gln	+0.03	No binding	
l-Gln	ND	No binding	
***N*-Phthaloyl-l-Glu**	+6.23	No binding	

a Compounds that resulted in a >2°C shift in melting temperature are in boldface type. ND, not determined.

**FIG 3:**
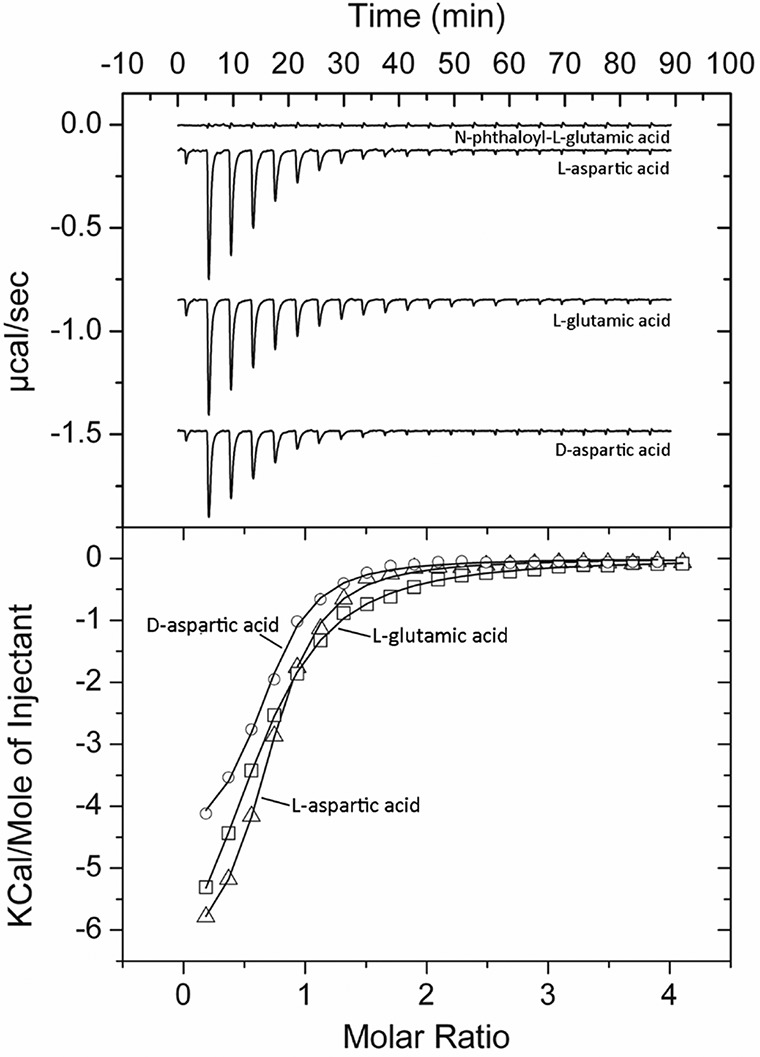
Microcalorimetric studies showing the binding of different d- and l-amino acids to PsPto-PscA-LBD. (Top) Titration raw data for the injection of 8 μl of 0.5 to 1 mM ligand solutions into 15 μM PsPto-PscA-LBD. (Bottom) Integrated, dilution heat-corrected, and concentration-normalized peak areas fitted with the one-binding-site model of ORIGIN.

To derive the thermodynamic binding parameters, the protein was analyzed by isothermal titration calorimetry (ITC). The titration of PsPto-PscA-LBD with the l- and d-enantiomers of Asp and l-Glu produced significant exothermic heat changes that diminished as titration proceeded (Fig. 3). Data analysis revealed that l- and d-Asp bound to PsPto-PscA-LBD with the same affinity of 1.2 ± 0.1 μM (Table 1), whereas l-Glu bound with a slightly lower affinity (*K_D_* [equilibrium dissociation constant] = 3.4 ± 0.1 μM). No binding heats were observed for *N*-phthaloyl-l-glutamate (Fig. 3). Since ITC permits visualization of only high-affinity binding events, it cannot be excluded that this compound binds to the protein with a much lower affinity. We also conducted ITC measurements with several related amino acids that did not cause significant *T_m_* shifts (Table 1). In all cases, an absence of binding was noted, indicating that PsPto-PscA-LBD specifically binds l- and d-Asp and l-Glu. These results are in agreement with those observed for the P. syringae pv. actinidiae homologue PscA (38). Finally, microcalorimetric titrations with l-tartrate, a d-Asp homologue abundant in plants, did not reveal binding (see Fig. S1A in the supplemental material).

10.1128/mBio.01868-19.2 FIG S1 (A) Microcalorimetric titration of 30 μM PsPto-PscA-LBD with 500 μM solutions of l-aspartate and l-tartrate. (Top) Raw titration data for the injection of 8-μl aliquots of the ligand solution into protein. (Bottom) Concentration-normalized and dilution heat-corrected integrated peak areas of the titration with l-aspartate. The continuous line is the best fit using the “one-binding-site” model of the MicroCal version of ORIGIN. (B) Quantitative assays of capillary chemotaxis of P. syringae pv. tomato (WT) and the PsPto-*pscA* mutant toward tartrate. The data have been corrected with the number of cells that swam into buffer-containing capillaries. Shown are means and standard errors from three independent experiments conducted in triplicate. ANOVA was performed, followed by Fisher’s LSD test (*, *P < *0.05). Download FIG S1, TIF file, 0.2 MB. Copyright © 2019 Cerna-Vargas et al. 2019 Cerna-Vargas et al. This content is distributed under the terms of the Creative Commons Attribution 4.0 International license.

### PsPto-PscA ligands mediate chemotaxis.

**FIG 4:**
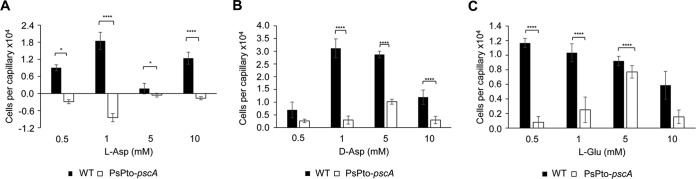
Quantitative assays of capillary chemotaxis of P. syringae pv. tomato (WT) and the PsPto-*pscA* mutant toward l-Asp (A), d-Asp (B), and l-Glu (C). The data have been corrected with the number of cells that swam into buffer-containing capillaries. Shown are means and standard errors from three independent experiments conducted in triplicate. Generalized linear models (GzLMs) were performed, followed by Fisher’s least significant difference (LSD) test (*, *P < *0.05; **, *P < *0.01; ***, *P < *0.005; ****, *P < *0.001), with the exception of l-Asp at 0.5 mM and l-Asp at 5 mM, where ANOVA was performed, followed by Fisher’s LSD test (*, *P < *0.05).

To investigate the chemotactic response of P. syringae pv. tomato to the three PsPto-PscA ligands, we conducted quantitative capillary chemotaxis assays with the WT (wild-type) strain. Amino acids were used at concentrations ranging from 0.5 to 10 mM. We observed significant responses toward l- and d-Asp and l-Glu, with maxima at 1 mM and 0.5 mM, respectively (Fig. 4). It was reported previously that l-Asp and l-Glu are used by P. syringae pv. tomato as carbon and nitrogen sources (49), a finding that we have confirmed (Fig. S2A and B). In contrast, d-Asp is not used as a nutrient source (Fig. S2E) by P. syringae pv. tomato and does not alter bacterial growth (Fig. S2F).

10.1128/mBio.01868-19.3 FIG S2 (A and B) Growth of P. syringae pv. tomato in minimal medium supplemented with l-Asp or l-Glu at 5 and 10 mM as the sole carbon (A) or nitrogen (B) source. (C and D) Growth of P. syringae pv. tomato in minimal medium supplemented with l-Arg or d-Glu at 5 and 10 mM as the sole carbon (C) or nitrogen (D) source. (E and F) Growth of P. syringae pv. tomato in minimal medium supplemented with d-Asp as the sole carbon or nitrogen source (E) and toxicity assay of d-Asp supplemented at different concentrations in KB and minimal media (F). Download FIG S2, TIF file, 0.4 MB. Copyright © 2019 Cerna-Vargas et al. 2019 Cerna-Vargas et al. This content is distributed under the terms of the Creative Commons Attribution 4.0 International license.

To determine whether these chemotactic responses are mediated by PsPto-PscA, we conducted assays with a mutant in which the *pscA* gene was insertionally inactivated. This mutant did not respond to l-Asp, whereas chemotaxis to d-Asp and l-Glu was significantly reduced (Fig. 4A), indicating that PsPto-PscA is the sole chemoreceptor for l-Asp, whereas additional receptors are likely to respond to the other two ligands (Fig. 4B and C).

Chemotaxis assays revealed that *pscA* provided in *trans* complemented the reduced chemotactic response of the *pscA* mutant toward l-Asp, d-Asp, and l-Glu (Fig. S3). Taken together, these results indicate that the chemoreceptor PsPto-PscA mediates chemotactic responses to d-Asp, l-Asp, and l-Glu.

10.1128/mBio.01868-19.4 FIG S3 Quantitative assay of capillary chemotaxis of P. syringae pv. tomato (WT), the PsPto-*pscA* mutant, and the complemented mutant (PsPto-*pscA-*Comp) toward l-Asp, d-Asp, and l-Glu at 1 mM. The data have been corrected with the number of cells that swam into buffer-containing capillaries. Shown are means and standard errors from three independent experiments conducted in triplicate. Generalized linear models (GzLMs) were performed, followed by Fisher’s least significant difference (LSD) test (*, *P < *0.05; **, *P < *0.01; ***, *P < *0.005; ****, *P < *0.001). Download FIG S3, TIF file, 0.1 MB. Copyright © 2019 Cerna-Vargas et al. 2019 Cerna-Vargas et al. This content is distributed under the terms of the Creative Commons Attribution 4.0 International license.

### Perception of PsPto-PscA ligands controls biofilm formation and swarming motility.

The regulatory mechanisms that govern biofilm dynamics are highly complex and remain poorly understood. Several studies highlight the involvement of chemosensory pathways in the biofilm formation process (12, 50, 51), and a role of specific chemoreceptors in biofilm formation has been reported for Pseudomonas putida KT2440 and P. aeruginosa (12, 52). Furthermore, d-amino acids were found to trigger biofilm disassembly in some bacteria (53, 54), while they had no effect on others (55).

**FIG 5:**
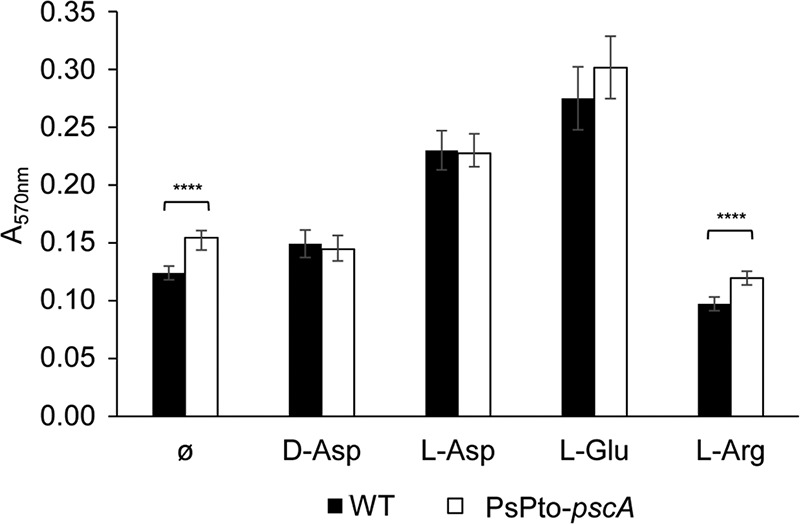
Biofilm formation in MGA medium. Total biofilm formation was quantified using the absorbance of crystal violet at 570 nm. Medium was supplemented with 0.5 mM d-Asp, 1 mM l-Glu, or 1 mM l-Asp. Shown are means and standard errors from at least three independent experiments conducted in triplicate. GzLM analysis was performed, followed by Fisher’s LSD test (****, *P < *0.001).

To investigate whether PsPto-PscA is involved in biofilm formation, we analyzed WT and mutant strains grown under static conditions during 24 h. A modest but significant increase in biofilm formation was observed for the PsPto-*pscA* mutant with respect to that of the WT strain (Fig. 5).

To assess the role of PsPto-PscA ligands in biofilm formation, we developed an assay in which saturating ligand concentrations were present, putatively causing complete receptor saturation, preventing a response. The WT strain showed increased biofilm formation in the presence of l- and d-Asp and l-Glu, reaching levels similar to those of the *pscA* mutant. In contrast, the addition of l-Arg, a compound that does not bind to PsPto-PscA, did not increase biofilm formation in the WT strain (Fig. 5).

**FIG 6:**
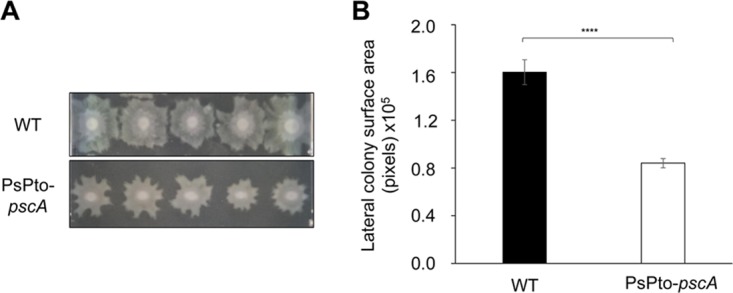
Impact of *pscA* mutation on swarming motility. Five replicates of each strain were placed onto a single plate and examined after 16 h. (A) Photographs of representative swarm colonies. (B) Quantification of the lateral colony surface area in digital images of the colonies. Shown are means and standard errors from three independent experiments. GzLM analysis was performed, followed by Student’s *t* test (****, *P < *0.001).

Considering the increased biofilm formation of the *pscA* mutant, and the inverse regulation of biofilm formation and swarming motility in *Pseudomonas* (35, 56), we assessed bacterial swarming of the WT and mutant strains after 16 h. Inactivation of *pscA* reduced swarming motility compared to the WT (Fig. 6).

### Intracellular levels of c-di-GMP are increased in the PsPto-*pscA* mutant.

**FIG 7:**
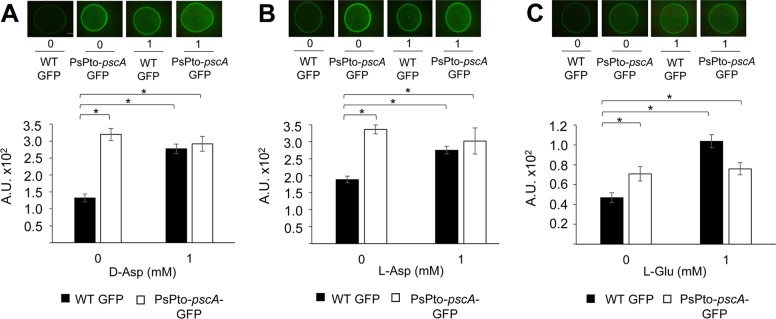
Effect of PsPto-*pscA* ligands on c-di-GMP levels. Fluorescence intensities of strains harboring the c-di-GMP biosensor plasmid pCdrA::*gfp^S^* grown in M9 medium supplemented with d-Asp (A), l-Asp (B), and l-Glu (C) were determined. Shown are means and standard errors from three independent experiments. ANOVA was performed, followed by Fisher’s LSD test (*, *P < *0.05). A.U., arbitrary units.

The role of c-di-GMP in the transition between motile and sessile lifestyles has been well documented in many bacteria (57–59) and was also found associated with the inverse regulation of biofilm formation and swarming in species like P. aeruginosa (60, 61), Vibrio parahaemolyticus (62), and P. syringae pv. tomato (34). We therefore quantified c-di-GMP levels in the WT and *pscA* mutant strains by introducing the plasmid pCdrA::*gfp^S^* (Text S1 and Table S1), which harbors a transcriptional fusion of the c-di-GMP-responsive *cdrA* promoter to a gene encoding green fluorescent protein (63). The fluorescence intensity of the reporter correlates with the intracellular c-di-GMP levels. As a positive control, we used the pJBpleD* plasmid, which confers high levels of c-di-GMP (64). The resulting strains were grown on agar plates for 24 h and subsequently analyzed by fluorescence microscopy, showing that the fluorescence intensity was significantly higher in the *pscA* mutant than in the WT strain (Fig. 7A to C). The complementation of the mutant with the *pscA* gene resulted in reduced c-di-GMP levels (Fig. S4). The increased c-di-GMP level in the mutant strain is in accordance with its enhanced biofilm formation (Fig. 5) and the reduced swarming phenotype (Fig. 6). Moreover, the addition of saturating concentrations of PsPto-PscA ligands caused an increase in the c-di-GMP levels in the WT strain on an order similar to that found in the mutant strain (Fig. 7A to C).

10.1128/mBio.01868-19.1 TEXT S1 Supplemental materials and methods. Download Text S1, DOCX file, 0.02 MB. Copyright © 2019 Cerna-Vargas et al. 2019 Cerna-Vargas et al. This content is distributed under the terms of the Creative Commons Attribution 4.0 International license.

10.1128/mBio.01868-19.5 FIG S4 Fluorescence intensity of PsPto strains harboring the c-di-GMP biosensor plasmid pCdrA::*gfp^S^.* Fluorescence was quantified as the corrected total cell fluorescence. A.U, arbitrary units. Experiments were conducted in M9 minimal medium. Shown are means and standard errors from three independent experiments. ANOVA was performed, followed by Fisher’s LSD test (*, *P < *0.05). Download FIG S4, TIF file, 0.2 MB. Copyright © 2019 Cerna-Vargas et al. 2019 Cerna-Vargas et al. This content is distributed under the terms of the Creative Commons Attribution 4.0 International license.

10.1128/mBio.01868-19.9 TABLE S1 Bacteria and plasmids used. Download Table S1, DOCX file, 0.02 MB. Copyright © 2019 Cerna-Vargas et al. 2019 Cerna-Vargas et al. This content is distributed under the terms of the Creative Commons Attribution 4.0 International license.

### A PsPto-*cheA2* mutant is defective in chemotaxis but shows increased c-di-GMP levels.

**FIG 8:**
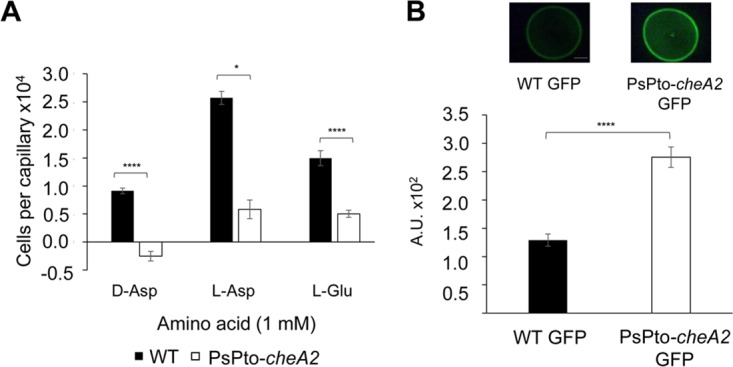
Effect of PsPto-PscA ligands on chemotaxis and c-di-GMP levels in a *cheA2* mutant. (A) Quantitative capillary chemotaxis assay of P. syringae pv. tomato (WT) and the *cheA2* mutant (PsPto-*cheA2*). (B) Fluorescence intensity of strains harboring the c-di-GMP biosensor plasmid pCdrA::*gfp^S^.* Shown are means and standard errors from three independent experiments. GzLM analysis was performed, followed by Student’s *t* test (****, *P < *0.001).

In order to identify the chemotaxis pathway of PsPto-PscA, we constructed a mutant strain of the homologue of P. aeruginosa PAO1 *cheA* (*PA1458*), previously described to be involved in the chemotaxis pathway of many MCPs. This gene (*PSPTO_1982* or *cheA2*) is located in P. syringae pv. tomato chemotaxis cluster I (65), and the encoded protein shares 78% sequence identity with P. aeruginosa PAO1 CheA (Fig. S5). In P. aeruginosa, the core proteins of the chemotaxis pathway are encoded by two clusters, namely, clusters I and V. Cluster V contains the CheR and CheV proteins, and this cluster is also found in P. syringae pv. tomato (Fig. S5). The PsPto-*cheA2* mutant lost the chemotactic response to the three ligands of PsPto-PscA. This mutant also showed significantly higher c-di-GMP levels than the WT strain (Fig. 8). In order to ascertain whether PsPto-CheA2 is the autokinase involved in chemotaxis, we measured responses to serine, spermidine, and succinic acid. Our results showed that the PsPto*-cheA2* mutant lost the ability for chemotaxis to the three compounds tested (Fig. S6).

10.1128/mBio.01868-19.6 FIG S5 Comparison of chemotaxis clusters I and V of PAO1 and P. syringae pv. tomato. Shown is the organization of different genes according to the Pseudomonas database (Pseudomonas.com). CheA and CheR1 homologues are in green and yellow, respectively. Download FIG S5, TIF file, 0.2 MB. Copyright © 2019 Cerna-Vargas et al. 2019 Cerna-Vargas et al. This content is distributed under the terms of the Creative Commons Attribution 4.0 International license.

10.1128/mBio.01868-19.7 FIG S6 Quantitative assay of capillary chemotaxis of P. syringae pv. tomato (WT) and the *cheA2* mutant (PsPto-*cheA2*) toward l-Ser, spermidine, and succinate at 1 and 5 mM. The data have been corrected with the number of cells that swam into buffer-containing capillaries. Shown are means and standard errors from three independent experiments conducted in triplicate. Generalized linear models (GzLMs) were performed, followed by Fisher’s least significant difference (LSD) test (*, *P* < 0.05). Download FIG S6, TIF file, 0.1 MB. Copyright © 2019 Cerna-Vargas et al. 2019 Cerna-Vargas et al. This content is distributed under the terms of the Creative Commons Attribution 4.0 International license.

### PsPto-PscA function controls virulence of P. syringae pv. tomato in tomato plants.

**FIG 9:**
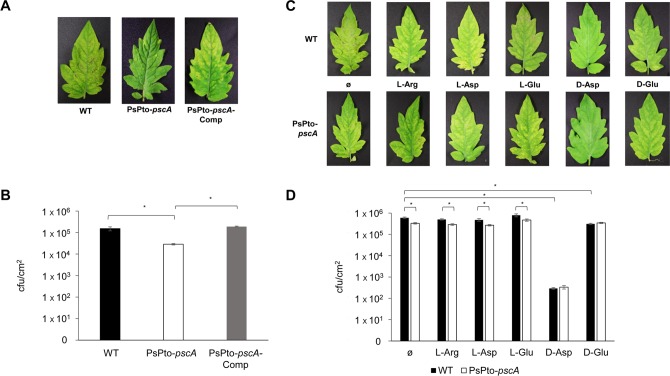
PsPto-PscA is required for the full virulence of P. syringae pv. tomato. (A) Virulence of P. syringae pv. tomato WT, mutant (PsPto-*pscA*), and complemented (PsPto-*pscA*-Comp) strains. (B) Plant colonization based on bacterial population sizes in tomato leaves at 6 days postinoculation, after spray inoculation of bacterial suspensions (10^8^ CFU/ml). Shown are means and standard errors from three independent experiments. ANOVA was performed, followed by Fisher’s LSD test (*, *P < *0.05). (C) Virulence of P. syringae pv. tomato (WT) and a PsPto-*pscA* mutant when the indicated amino acids were added to the bacterial suspension before infection. (D) Plant colonization based on bacterial population sizes in tomato leaves at 6 days postinoculation, after spray inoculation of bacterial suspensions (10^8^ CFU/ml), in the presence of the indicated amino acids. Shown are means and standard errors from at least three independent experiments. GzLM analysis was performed, followed by Fisher’s LSD test (****, *P < *0.001).

The decreased swarming motility and increased c-di-GMP levels of the PsPto-*pscA* mutant may potentially affect virulence. To test this hypothesis, we conducted virulence assays in which leaves of tomato plants were inoculated with the WT and mutant strains. At 6 days postinoculation, bacterial populations were quantified. Data showed significant reductions in both symptom development and bacterial populations for the PsPto-*pscA* mutant compared to the WT strain. A complemented PsPto-*pscA* mutant strain restored virulence to WT levels (Fig. 9A and B).

In subsequent studies, we analyzed bacterial pathogenicity in the presence of the PsPto-PscA ligands. Chemotaxis is based on a ligand gradient covering the chemoreceptor response range (6), and chemoreceptor saturation with the ligand will prevent taxis. We therefore conducted virulence assays in the presence and absence of saturating concentrations of d-Asp, l-Asp, and l-Glu. To this end, tomato leaves were spray inoculated with a bacterial suspension with or without 1 mM amino acid. As a control, we inoculated plants with bacteria containing 1 mM l-Arg or d-Glu, compounds to which P. syringae pv. tomato does not show chemotaxis (data not shown). After 6 days postinoculation, dramatic reductions in both symptom development and bacterial populations were observed for the plants inoculated with a bacterium–d-Asp mixture compared to plants inoculated without d-Asp (Fig. 9C and D). In contrast to the drastic decreases in symptom development and leaf colonization that occurred in the presence of d-Asp, only a slight reduction was observed when d-Glu was added to the inoculum. Moreover, the presence of l-Arg, l-Asp, and l-Glu did not alter disease development. As expected, the PsPto-*pscA* mutant strain was impaired in its virulence despite the addition of l-amino acids to the inoculum (Fig. 9C and D).

## DISCUSSION

Knowledge on the function of chemoreceptors in plant-pathogenic bacteria is very scarce. In this work, we have identified the ligands of the PsPto-PscA chemoreceptor and demonstrate that it exerts multiple functions. Apart from mediating chemotaxis, this receptor was shown to be involved in regulating c-di-GMP levels, which was reflected in associated phenotypic manifestations such as changes in biofilm formation or swarming motility. Furthermore, PsPto-PscA was also involved in controlling P. syringae pv. tomato virulence.

There is very substantive evidence that chemotaxis is a key requisite for efficient pathogenesis in plant pathogens (16). It is generally accepted that chemoeffectors released by either plant wounds or stomata induce chemotaxis toward these plant openings, providing access to the apoplast to initiate plant infection. Most of the studies available to date have analyzed mutants in *cheA* or flagellar genes that resulted in nonchemotactic or nonmotile phenotypes, respectively (16). However, information regarding particular bacterial chemoreceptors and the specific plant signals that they sense is scarce. Here, we show that the PsPto-PscA chemoreceptor binds specifically to d/l-Asp and l-Glu and that signaling through PsPto-PscA affects bacterial virulence in host plants. This ligand specificity is underlined by the observation that l-tartrate, a d-Asp homologue present in plants, failed to bind to PsPto-PscA, and hence, l-tartrate chemotaxis was not altered in the PsPto-*pscA* mutant (see Fig. S1B in the supplemental material). PsPto-PscA contains a dCACHE LBD; a significant number of dCACHE-containing chemoreceptors have been reported so far, and many of them are characterized by a broad ligand spectrum since they recognize almost all proteinogenic amino acids. Representative members of this family include the chemoreceptors PctA of P. aeruginosa (39, 40), McpU of Sinorhizobium meliloti (66), McpC of Bacillus subtilis (43), McpX of Vibrio cholerae (44), and CtaA and CtaB of Pseudomonas fluorescens (67). In marked contrast is PsPto-PscA, which has a very narrow ligand range and recognizes only three acidic amino acids. Interestingly, Asp and Glu are the most abundant proteinogenic amino acids in plants (68, 69) and specifically in the tomato apoplast (49). Moreover, the presence, although in smaller amounts, of d-amino acids in plants has also been reported (70) and was found to be due to the activity of plant racemases or to the uptake of bacterium-derived amino acids from soil.

It can therefore be hypothesized that the abundance of these compounds in tomato apoplasts has driven the evolution of a chemoreceptor that recognizes these compounds with high specificity and that their detection during plant infection is crucial for optimal infection. Aspartate, next to its abundance in apoplasts, appears to be an amino acid of particular relevance for bacteria since several other chemoreceptors have evolved that recognize this amino acid with high specificity. The Tar receptors of E. coli and Salmonella enterica serovar Typhimurium are the central model chemoreceptors to study chemotactic signaling. Both proteins were found to bind aspartate with high preference, and the obtained dissociation constants are very similar to those obtained in this study for PsPto-PscA (71–73). Tlp1 (renamed CcaA) of the human pathogen Campylobacter pylori is an aspartate-specific chemoreceptor (46, 74) and was found to play an important role in virulence since experimentation with the mutant strain in different hosts resulted in a number of pathological changes in infection experiments (75). Although the corresponding molecular mechanisms remain unclear, these data underline the importance of signaling mediated by aspartate-specific chemoreceptors.

The response to extracytoplasmic signals by bacteria is achieved mainly by two-component signaling systems (TCSs) and chemosensory pathways (76). Many bacteria possess multiple copies of TCSs and chemosensory pathways. Indeed, the genome of P. syringae pv. tomato encodes four chemosignaling pathways (Fig. S7). A central question in signal transduction research resides in establishing whether there is any functional cross talk between these multiple copies of homologous signal transduction systems. Cross talk between signal transduction pathways, while usually considered undesired in the evolution of protein-protein interactions, might be beneficial as a result of an evolutionary response to a given environmental situation. Although for TCSs, significant insight into this issue has been obtained, mainly by the pioneering work of the Laub laboratory (77), much less information is available for chemosensory pathways (12). We show here that mutation of PsPto-*pscA* increases biofilm formation (Fig. 5) and decreases swarming motility (Fig. 6), which are phenotypes most likely caused by the increase in c-di-GMP levels observed in this mutant (Fig. 7). Furthermore, mutation of PsPto-*cheA2* of the chemotaxis pathway generates a dramatic reduction in taxis to several compounds, including the PsPto-PscA ligands, and an increase in c-di-GMP levels. These data suggest that PsPto-CheA2 may interact with other MCPs and that stimulation of PsPto-CheA2 modulates c-di-GMP levels. The results also indicate that the chemotaxis pathway of P. syringae pv. tomato is not an insulated pathway but interacts with different signaling systems, such as the one governing biofilm formation.

10.1128/mBio.01868-19.8 FIG S7 Schematic representation of different gene clusters in P. syringae pv. tomato that encode chemosensory pathways. Shown in green is the cluster homologous to the P. aeruginosa Wsp pathway. Download FIG S7, TIF file, 0.2 MB. Copyright © 2019 Cerna-Vargas et al. 2019 Cerna-Vargas et al. This content is distributed under the terms of the Creative Commons Attribution 4.0 International license.

One of the four chemosensory pathways in P. syringae pv. tomato, encoded by cluster III (Fig. S7), is homologous to the P. aeruginosa Wsp pathway that was shown to modulate c-di-GMP levels in response to as-yet-unidentified environmental cues (10). Data obtained with P. aeruginosa indicate that a given chemoreceptor interacts specifically with only one CheA paralogue (78). In the present case, we hypothesize that PsPto-PscA likely interacts with CheA2, which, however, would signal to its cognate receptor CheY2 as well as to the CheY homologue of the Wsp pathway (WspR_Pspto). CheY2 of P. syringae pv. tomato is a receiver-domain-only response regulator that is likely to interact in its phosphorylated state with the flagellar motor causing taxis. WspR_Pspto is a fusion of a receiver domain with a GGDEF diguanylate cyclase domain. Studies in P. aeruginosa have shown that WspR phosphorylation alters the catalytic activity of its GGDEF domain (79).

Interference of chemotaxis has been proposed to be a strategy to fight bacterial pathogens (80). Chemotaxis is required for localizing to plant openings in order to enter the plant and establish bacterial infection. We show here that saturating PsPto-PscA with d-Asp reduced bacterial infection, which is most likely due to a reduction in chemotaxis toward plant openings. However, the addition of l-Asp and l-Glu did not cause any significant effect on virulence. This may seem to be contradictory at first sight but may be due to the fact that d-Asp cannot be metabolized, whereas both l-enantiomers are efficiently used as nutrients (Fig. S2). Metabolization of these compounds will lead to the formation of a compound gradient, which in turn induces an additional chemotactic response. One may argue that growth promotion by l-Asp and l-Glu may cancel out the negative effect that these ligands had on virulence (as observed with d-Asp). However, l-Arg, a compound that also stimulates P. syringae pv. tomato growth (Fig. S2D) but that is not a PsPto-PscA ligand, did not produce significant effects on virulence. This result indicates that although P. syringae pv. tomato can grow on l-amino acids, the corresponding increase in cell density is rather modest. Moreover, the addition of other d-amino acids like d-Glu, which is not a P. syringae pv. tomato chemoattractant, caused only minor reductions in symptom development and leaf colonization, which is in marked contrast to the severe effects observed in the presence of d-Asp. Taken together, these data show that d-Asp, under saturating conditions, reduces virulence in a specific manner. Therefore, the addition of nonmetabolizable chemoeffectors may be an alternative to inhibit bacterial entry into plants.

## MATERIALS AND METHODS

### Bacterial strains, culture media, and growth conditions.

Bacterial strains and plasmids used in this work are listed in Text S1 and Table S1 in the supplemental material. P. syringae pv. tomato DC3000 and its derivative strains were grown at 28°C in KB (King's B) medium (81). E. coli derivatives were grown at 37°C in LB medium (82). When appropriate, the following antibiotics were added to the medium at the following concentrations: rifampin at 25 μg/ml, streptomycin at 50 μg/ml, kanamycin at 25 μg/ml, ampicillin at 100 μg/ml, chloramphenicol at 10 μg/ml, nalidixic acid at 10 μg/ml, and gentamicin at 5 μg/ml.

### Identification and classification of MCP signal domains.

The search for the MCP signal domains (PF00015) was performed using an *ad hoc* pipeline as described previously by Río-Álvarez et al. (83). Transmembrane (TM) domains were identified individually using the TMHMM server v. 2.0 (http://www.cbs.dtu.dk/services/TMHMM/) and the DAS transmembrane region prediction algorithm (84). Each MCP was analyzed for Pfam matches (https://pfam.xfam.org/) and categorized according to its LBD. LBDs were considered the domains different from the MCP signal (PF00015) and HAMP (domain present in histidine kinases, adenylyl cyclases, methyl-accepting proteins, and phosphatases) (PF00672).

### Construction of the expression plasmid for PsPto-PscA-LBD.

A complete list of plasmids used in this study is available in Table S1, and a complete list of primers can be found in Table S2.

10.1128/mBio.01868-19.10 TABLE S2 Primers used. Download Table S2, DOCX file, 0.01 MB. Copyright © 2019 Cerna-Vargas et al. 2019 Cerna-Vargas et al. This content is distributed under the terms of the Creative Commons Attribution 4.0 International license.

The DNA fragment encoding the PSPTO_2480 (amino acids 30 to 278) LBD was amplified by PCR using primers 2480LBDFw and 2480LBDRv (Table S2), and the resulting PCR fragment was finally cloned into the pDEST17 expression vector using Gateway technology (Invitrogen, CA, USA). The verified resulting plasmid, p2480-LBD, was transformed into E. coli BL21(DE3). A detailed description of the construction can be found in Text S1.

### Overexpression and purification of PsPto-PscA-LBD.

E. coli BL21(DE3) containing p2480-LBD was grown in 2-liter Erlenmeyer flasks containing 400 ml LB medium supplemented with 100 μg/ml ampicillin at 30°C. Once the culture reached an optical density at 600 nm (OD_600_) of 0.5, protein overexpression was induced by the addition of 0.5 mM isopropyl-β-d-1-thiogalactopyranoside (IPTG). Growth was then continued at 18°C overnight prior to cell harvest by centrifugation at 6,000 × *g* for 20 min at 4°C. Cell pellets were resuspended in buffer A (30 mM Tris-HCl, 300 mM NaCl, 10 mM imidazole, 10% [vol/vol] glycerol [pH 8.0]) and broken by French press treatment at a gauge pressure of 62.5 lb/in^2^. After centrifugation at 20,000 × *g* for 1 h, the supernatant was loaded onto a 5-ml HisTrap column (Amersham Biosciences) previously equilibrated with 5 column volumes of buffer A, washed with buffer A containing 40 mM imidazole, and eluted with a linear gradient of 40 to 500 mM imidazole in buffer A. Protein-containing fractions were pooled and dialyzed into HNG buffer (50 mM HEPES, 300 mM NaCl, 10% [vol/vol] glycerol [pH 8.0]) for immediate analysis.

### Thermal shift assay-based high-throughput ligand screening.

Thermal shift assays were performed on a Bio-Rad MyIQ2 real-time PCR instrument. Ligands from the compound array (Biolog, Hayward, CA, USA) were dissolved in 50 μl of Milli-Q water, which, according to the manufacturer, corresponds to a concentration of 10 to 20 mM. Screening was performed using 96-well plates. Assay mixtures (25 μl) contained 15 μM protein dialyzed into HNG buffer, SYPRO orange (Life Technologies) at a 5× concentration, and ligands at final concentrations of 1 to 2 mM. In a single well (ligand-free protein), the compound was replaced by water. Samples were heated from 23°C to 85°C at a scan rate of 1°C/min. The protein unfolding curves were obtained by monitoring the changes in SYPRO orange fluorescence. Melting temperatures were determined using the first derivative values from the raw fluorescence data.

### Isothermal titration calorimetry binding studies.

Experiments were conducted on a VP microcalorimeter (MicroCal, Amherst, MA, USA) at 25°C. PsPto-PscA-LBD was dialyzed overnight against HNG buffer, adjusted to a concentration of 15 μM, and placed into the sample cell of the instrument. The protein was titrated by the injection of 8-μl aliquots of 0.5 to 1 mM ligand solutions that were prepared in HNG buffer immediately before use. The mean enthalpies measured from the injection of ligands into buffer were subtracted from raw titration data prior to data analysis with the MicroCal version of ORIGIN. Data were fitted with the “one-binding-site” model.

### Construction of mutants.

The generation of PsPto*-pscA* and PsPto-*cheA2* mutant strains was carried out by single-crossover integration. A detailed description can be found in Text S1.

### Quantitative capillary chemotaxis assays.

Cultures grown overnight were diluted to an OD_600_ of 0.05 in KB buffer and grown at 28°C with orbital shaking. At the early stationary phase of growth, cultures were centrifuged at 1,750 × *g* for 5 min, and the resulting pellet was washed twice with 10 mM HEPES (pH 7.0). Cells were resuspended in HEPES and adjusted to an OD_600_ of 0.25. Next, 230-μl samples were placed into each well of a 96-well plate. One-microliter capillaries were filled with the compound to be tested, immersed into the bacterial suspension, and incubated for 30 min. Capillaries were removed from the bacterial suspension and rinsed with sterile water, and the content was expelled into 1 ml of NB medium (1 g yeast extract, 2 g beef extract, 5 g NaCl, and 5 g Bacto peptone [per liter]). Serial dilutions were plated onto NB medium with the appropriate antibiotics, and the number of CFU was determined. In all cases, data were corrected by subtracting the number of cells that swam into buffer-containing capillaries.

### Biofilm formation.

Biofilm formation assays were performed as described previously by Chakravarthy et al. (36), with a modified MG liquid medium, MGA (54 mM mannitol, 3.6 mM KH_2_PO_4_, 23 mM NaCl, 0.8 mM MgSO_4_, 18 mM NH_4_Cl [pH 7.0]). A detailed description can be found in Text S1.

### Swarming motility assays.

P. syringae pv. tomato strains were grown at 28°C for 24 h on KB agar. Cells were resuspended in KB medium to an OD_600_ of 1. Five microliters of the bacterial suspension was spotted onto soft KB agar (0.5% [wt/vol] agar). Plates were incubated for 16 h at 28°C at 80% relative humidity (RH) under dark conditions. Swarm colonies were photographed, and the surface area of each colony was quantified using the area selection tool of Adobe Photoshop software with readings in pixels.

### Colony-based c-di-GMP reporter assays.

Fluorescence intensity analyses using the c-di-GMP biosensor pCdrA::*gfp^S^* were carried out according to methods described previously by Corral-Lugo et al. (52), with slight modifications. A detailed description can be found in Text S1.

### Tomato virulence assays.

P. syringae pv. tomato strains were grown at 28°C for 24 h on KB agar in darkness. Cells were resuspended in 10 mM MgCl_2_ and diluted to 10^8^ CFU/ml. Three-week-old tomato plants (Solanum lycopersicum cv. Moneymaker) were sprayed with a suspension containing 10^8^ CFU/ml. Silwet L-77 was added to the bacterial suspensions at a final concentration of 0.02% (vol/vol). Where indicated, amino acids were added to a final concentration of 1 mM.

Plants were incubated in a growth chamber at 25°C at 60% RH with a daily light period of 12 h. Six days after inoculation, the leaf symptoms were recorded, and bacterial populations from three plants were measured by sampling five 1-cm-diameter leaf disks per plant. The infected leaf disks were washed twice with 10 mM MgCl_2_ prior to homogenization to eliminate the bacteria from the leaf surface. Plant material was homogenized in 10 mM MgCl_2_ and drop plated onto KB agar supplemented with the appropriate antibiotics. The average number of bacteria per square centimeter isolated from five infected tomato leaves was determined based on log-transformed data.

### Statistical analysis.

Differences among strains were compared using generalized linear models (GzLMs) when variances were different or one-way analysis of variance (ANOVA) when variances were equal, followed by Fisher’s least significant difference (LSD) *post hoc* test for multiple comparisons, performed using the statistical software package SPSS 22.0 (SPSS Inc., Chicago, IL, USA).
