# Supplementary figures and images for: Chemoperception of Specific Amino Acids Controls Phytopathogenicity in Pseudomonas syringae pv. tomato
Source: mBio. 2019 Oct 1;10(5):e01868-19. doi: 10.1128/mBio.01868-19 (PMC6775455; doi:10.1128/mBio.01868-19)

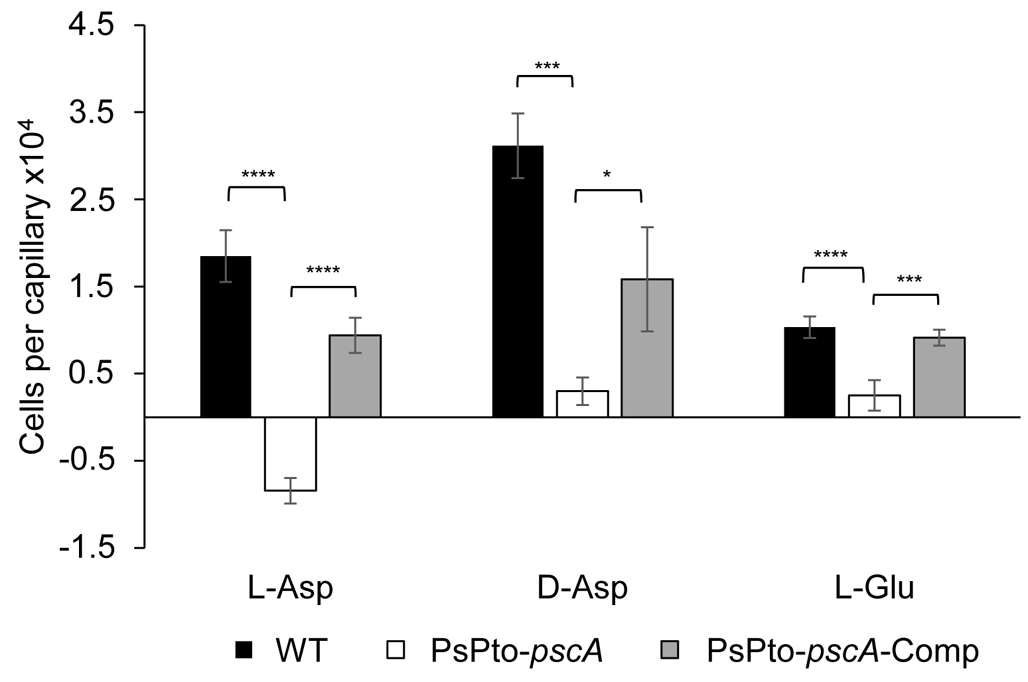

Supplement: FIG S3 [file mBio.01868-19-sf003.tif]

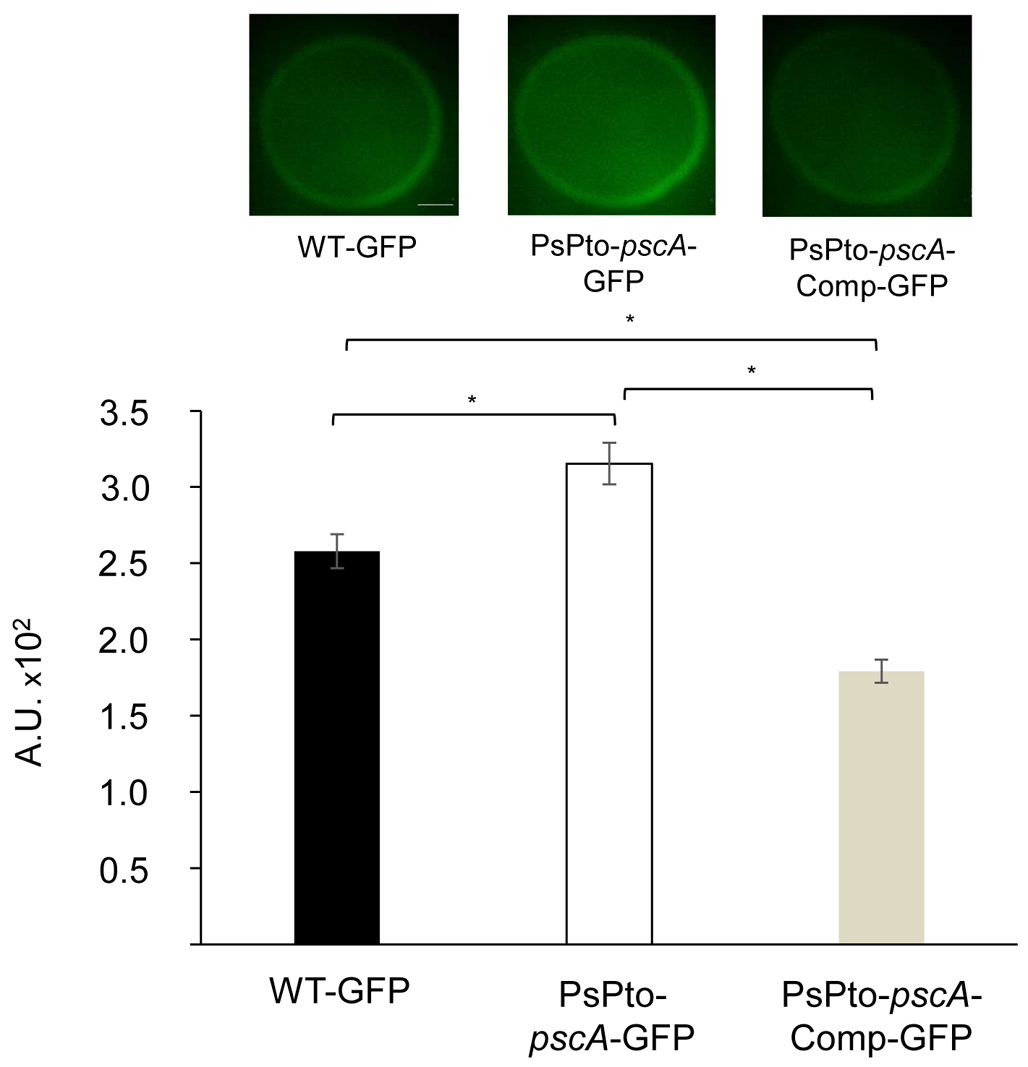

Supplement: FIG S4 [file mBio.01868-19-sf004.tif]

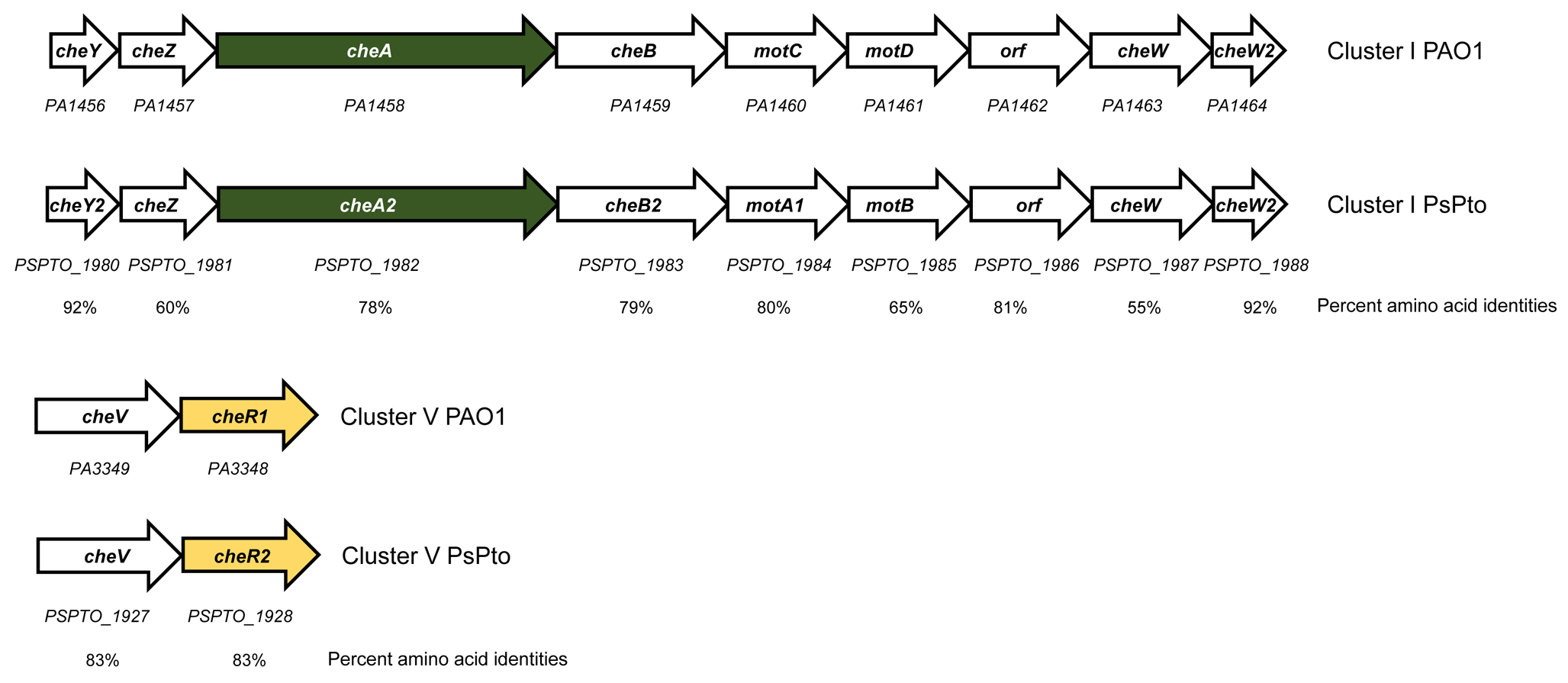

Supplement: FIG S5 [file mBio.01868-19-sf005.tif]

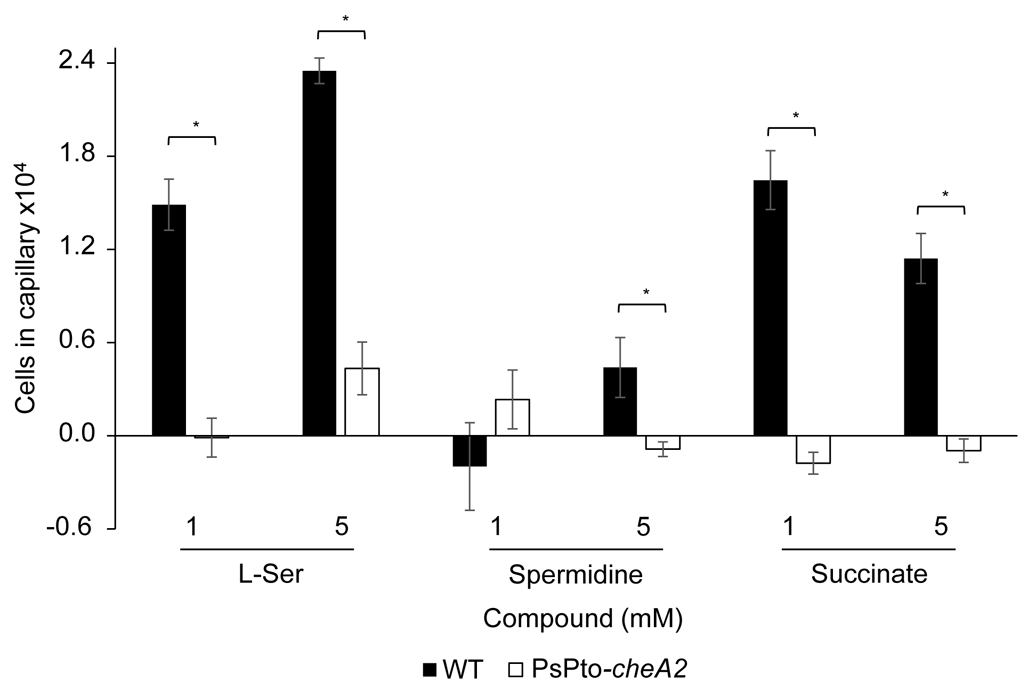

Supplement: FIG S6 [file mBio.01868-19-sf006.tif]

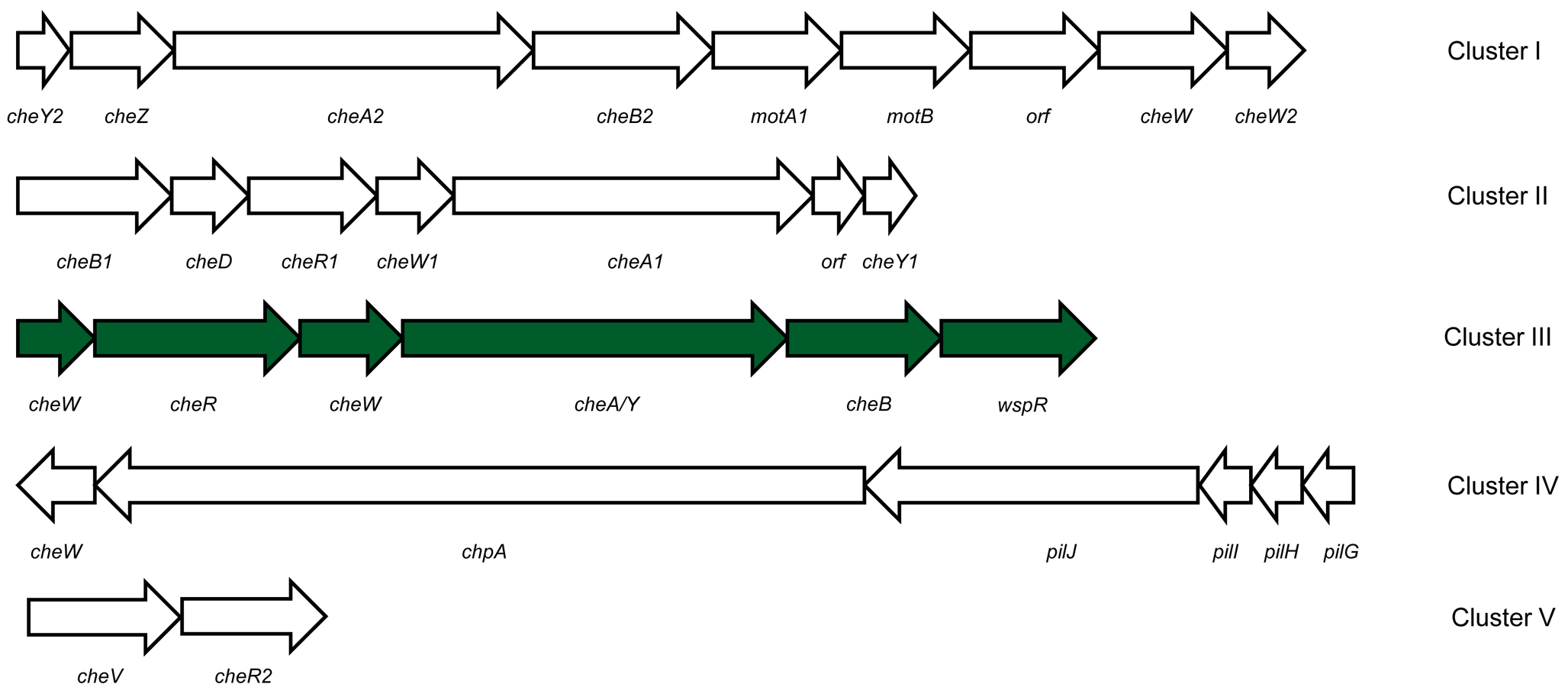

Supplement: FIG S7 [file mBio.01868-19-sf007.tif]
