# Supplementary material for: Chemoperception of Specific Amino Acids Controls Phytopathogenicity in Pseudomonas syringae pv. tomato
Source: mBio. 2019 Oct 1;10(5):e01868-19. doi: 10.1128/mBio.01868-19 (PMC6775455; doi:10.1128/mBio.01868-19)
Supplement: TEXT S1 [file mBio.01868-19-s0001.docx]

**Text S1. Supplemental Materials and Methods**

Content of Text S1

1. Supplemental Material and Methods
2. Construction of the expression plasmid for PsPto-PscA-LBD
3. Construction of mutants
4. Carbon and nitrogen utilization and toxicity assays
5. Biofilm formation
6. Colony based c-di-GMP reporter assays
7. References
8. **Supplemental Material and Methods**
9. **Construction of the expression plasmid for PsPto-PscA-LBD**

A complete list of plasmids used in this study is available in Text S1-Table 1 and a complete list of primers can be found in Text S1-Table 2.

The LBD of PSPTO_2480 (amino acids 30-278) was defined as the region between its two predicted transmembrane regions. The DNA fragment encoding its LBD was amplified by PCR using primers 2480LBDFw and 2480LBDRv (Text S1-Table), and the resulting PCR fragment was cloned into pENTR™/SD/D-TOPO vector using the Gateway TOPO cloning kit (Invitrogen, CA, USA). Positive colonies were confirmed by sequencing. This DNA construct was used to generate the expression clone through recombination with the pDEST™17 expression vector using the LR clonase™ II Enzyme Mix (Invitrogen, CA, USA). The resulting plasmid p2480-LBD was verified by PCR, restriction analysis and sequencing prior to the transformation into E. coli BL21 (DE3).

1. **Construction of mutants**

To generate the PsPto-pscA mutant strain, an internal 484-pb fragment of PSPTO_2480 (corresponding to nucleotides 72-556 of the coding sequence) was amplified by PCR using primers 2480XmaIFw and 2480XmaIRv (Text S1-Table 2). To generate the PsPto-cheA2 mutant strain, an internal 751-pb fragment of PSPTO_1982 (corresponding to nucleotides 125-876 of the coding sequence) was amplified by PCR using primers 1982XmaIFw and 1982XmaIRv (Text S1-Table 2). All primers contained XmaI restriction sites. The PCR products were cloned into pGEM-T easy vectors and transformed into E. coli DH5α. Positive colonies were digested with XmaI and the inserts were cloned into pKOSac101. The resulting plasmids, pKOSac101-2480 and pKOSac101-1982, were introduced into PsPto by electroporation according to Choi et al. (1). Plasmid integration was confirmed by PCR and Southern Blotting.

To complement the pscA mutation, the PSPTO_2480 gene and a 200-bp region upstream were amplified using primers 2480CompFw and 2480CompRv, (Text S1-Table 2), both containing an EcoRI restriction site. The PCR product was cloned into the EcoRI site of pBBR1MCS-2 (2). The resulting plasmid, pBBR1MCS-2-2480 was verified by DNA sequencing and transformed into PsPto-pscA mutant strain by electroporation, generating the complemented strain PsPto-pscA-Comp.

1. **Carbon and nitrogen utilization and toxicity assays**

A 1.5 mL aliquot of overnight-grown bacteria was washed twice with KB and then resuspended in 2 mL KB. After 2 h incubation with shaking at 28 ᵒC, cells were centrifuged and resuspended in two-fold M9 that lacked either ammonium chloride or glucose to a final OD_600_ of 0.28. Then, 75 µl aliquots were inoculated into microplate wells containing 75 µl the compound to test. For D-Asp toxicity assays, bacteria were centrifuged and resuspended in either M9 (supplemented with glucose 0.4% (w/v)) or KB, and toxicity was tested at 0.5, 1 and 5 mM concentrations.

1. **Biofilm formation**

Overnight cultures were washed three times in MGA liquid medium (54 mM mannitol, 3.6 mM KH2PO4, 23 mM NaCl, 0.8 mM MgSO4, 18 mM NH4Cl; pH 7.0) supplemented with 2 mM CaCl2 and adjusted to final OD600 = 0.02. 100 µl of bacterial suspensions, supplemented with the amino acid (1 mM L-Asp, L-Glu and L-Arg and 0.5 mM D-Asp) when corresponding, were added to 96-well plates and incubated at 28 °C for 24 h. After 24 h, planktonic cells were removed from wells and wells were gently washed 3 times with distilled water. 150 µl of 0.1% (w/v) crystal violet stain was added to each well and incubated for 30 min. After three gentle washes with distilled water, stained biofilms were resuspended in 30% (v/v) acetic acid. The A570 was recorded for each well and the average and standard deviation for technical replicates were computed.

1. **Colony-based c-di-GMP reporter assays**

Fluorescence intensity analyses using the c-di-GMP biosensor pCdrA::gfpS were carried out according to Corral-Lugo et al. (3) with slight modifications. Briefly, the reporter plasmid pCdrA::gfpS was transformed into PsPto wild-type, PsPto-pscA and PsPto-PscA-Comp strains by electroporation according to Choi et al. (1). Overnight bacterial cultures of the strains grown in M9 minimal medium (supplemented with 0.1 mM CaCl2, 2 mM MgSO4, 0.2% (w/v) citrate and 1 mM amino acid when corresponding) were adjusted to an OD600 of 0.05 and 20 µL drops were spotted on M9 agar plates supplemented with 1 mM of each amino acid separately containing the appropriate antibiotics. After 24 h incubation at 28 °C, colony morphology and fluorescence intensity were analyzed in a Leica MZ10F stereo microscope (Leica Microsystems, Wetzlar, Germany). Fluorescence was visualized employing a GFP filter set (emission/excitation filter 510/460 nm). Pictures were taken using Leica Application Suite software V4.3. Fluorescence was quantified as the corrected total cell fluorescence and expressed as A.U. (arbitrary Units) measured using ImageJ, specifically the Fiji distribution (4).

1. **References**

1. Choi KH, Kumar A, Schweizer P. 2006. A 10-min method for preparation of highly electrocompetent *Pseudomonas aeruginosa* cells: application for DNA fragment transfer between chromosomes and plasmid transformation. J Microbiol Methods 64:391-397.

2. Kovach ME, Elzer PH, Hill DS, Robertson GT, Farris MA, Roop RM, Peterson KM. 1995. Four new derivatives of the broad-host-range cloning vector pBBR1MCS carrying different antibiotic-resistance cassettes. Gene 166:175-176.

3. Corral-Lugo A, de la Torre J, Matilla MA, Fernández M, Morel B, Espinosa-Urgel M, Krell T. 2016. Assessment of the contribution of chemoreceptor-based signaling to biofilm formation. Environ Microbiol 18:3355-3372.

4. Schindelin J, Arganda-Carreras I, Frise E, Kaynig V, Longair M, Pietzsch T, Preibisch S, Rueden C, Saalfeld S, Schmid B, Tinevez J-Y, White DJ, Hartenstein V, Eliceiri K, Tomancak P, Cardona A. 2012. Fiji: an open-source platform for biological-image analysis. Nat Methods 9:676-682.
