# Supplementary material for: Chemoperception of Specific Amino Acids Controls Phytopathogenicity in Pseudomonas syringae pv. tomato
Source: mBio. 2019 Oct 1;10(5):e01868-19. doi: 10.1128/mBio.01868-19 (PMC6775455; doi:10.1128/mBio.01868-19)
Supplement: TABLE S1 [file mBio.01868-19-st001.docx]

| **Table S1.** Bacteria and plasmids used. | | |
| --- | --- | --- |
| Strains and plasmids | Relevant characteristics^a^ | Reference or source |
| ***E. coli*** |  |  |
| CC118λ*pir* | Sp^r^, ∆(*ara-leu*) *araD* ∆*lacX*74 *galE galK phoA*20 *thi*-1 | (*1*) |
|  | *rps* *rpoB argE recA*1 lysogenized with λ*pir* phage |  |
| DH5α | *supE*44 *lacU*169 (*Ø80lacZ*Δ M15) *hsdR*17 (r_k_-m_k_-) | (*2*) |
|  | *recA*1 *endA*1 *gyrA*96 *thi*-1 *relA*1 |  |
| BL21 (DE3) | *F^−^, ompI, hsdSB (*r_B_-m_B_-*) gal, dam, met* | (*3*) |
| ***P. syringae* pv. tomato DC3000** |  |  |
| PsPto | Rif^r^, wild type | (*4*) |
| PsPto*-pscA* | *PSPTO2480ins*::pKNG101 Sm^r^ derivative of PsPto | This study |
| PsPto-*pscA*-Comp | *PSPTO2480*::pBBR1-MCS2 derivative of *2480,* Km^r^, Km^r^ | This study |
| PsPto*-cheA2* | *PSPTO1982ins*::pKNG101 Sm^r^ derivative of PsPto | This study |
| WT-GFP | pCdrA*::gfp^s^* derivative of PsPto, Ap^r^, Gm^r^ | This study |
| PsPto*-pscA-*GFP | pCdrA*::gfp^s^* derivative of PsPto*-pscA*, Sm^r^, Ap^r^, Gm^r^ | This study |
| PsPto-*pscA*-Comp-GFP | pCdrA*::gfp^s^* derivative of PsPto-*i*-Comp, Sm^r^, Km^r^, Ap^r^, Gm^r^ | This study |
| PsPto*-cheA2*-GFP | pCdrA*::gfp^s^* derivative of PsPto*-cheA2*, Sm^r^, Ap^r^, Gm^r^ |  |
| PleD* | pJB3TcPleD* derivative of PsPto, Tc^r^, Ndx^r^ | This study |
| PleD*-GFP | pCdrA*::gfp^s^* derivative of PleD*, Tc^r^, Ndx^r^, Ap^r^, Gm^r^ | This study |
| **Plasmids** |  |  |
| pGEM-T easy® | Ap^r^ | Invitrogen |
| pGEM-T easy-2480 | Ap^r^ | This study |
| pGEM-T easy-1982 | Ap^r^ | This study |
| pKOSac101 | Sm^r^*; oriR6K mob* pKNG101 | This study |
|  | harboring an 144-bp internal deletion of *sacB* |  |
| pKOSac101-2480 | Sm^r^, pKNG101 with a 501-bp PCR fragment of *PSPTO_2480* cloned at XmaI sites | This study |
| pKOSac101-1982 | Sm^r^, pKNG101 with a 751-bp PCR fragment of *PSPTO_1982* cloned at XmaI sites | This study |
|  |  |  |
| pENTR^TM^/SD-TOPO | Entry vector for Gateway cloning, Km^r^ | Invitrogen, CA, USA |
| pENTR^TM^/SD-TOPO-2480 | Entry vector of Gateway cloning encoding LBD of PSPTO_2480, Km^r^ | This study |
| pDEST^TM^17 | N-terminal His-tagged protein expression vector, Ap^r^ | Invitrogen, CA, USA |
| p2480-LBD | N-terminal His-tagged PSPTO_2480-LBD expression vector, Ap^r^ | This study |
| pBBR1MCS-2 | Km^r^, *ori*RK2 *mob*RK2 | (*5*) |
| pBBR1MCS-2-2480 | Km^r^; a 2.1-kb PCR fragment containing the *PSPTO_2480* gene and its promoter region | This study |
| pJBpleD* | Ndx^r^, Tc^r^, pJB3Tc19 derivative bearing a 1,423-bp XbaI/EcoRI fragment containing *pleD** | (*6*) |
| pCdrA::*gfp*^S^ | pUCP22Not-P_cdrA_-RBS-CDS-RNaseIII-*gfp(*Mut3)-T_0_-T_1_, Ap^r^, Gm^r^ | (*7*) |
| Sm: streptomycin, Km: kanamycin, Ap; ampicillin, Gm: gentamicin, Tc: tetracycline, Ndx: nalidixic acid, | | |

**References**

1. Herrero M, de Lorenzo V, Timmis KN. 1990. Transposon vectors containing non-antibiotic resistance selection markers for cloning and stable chromosomal insertion of foreign genes in gram-negative bacteria. J Bacteriol 172: 6557-6567.

2. Hanahan D. 1983. Studies on transformation of *Escherichia coli* with plasmids. J Mol Biol 166: 557-580.

3. Studier FW, Moffatt BA. 1986. Use of bacteriophage T7 RNA polymerase to direct selective high-level expression of cloned genes. J Mol Biol 189:113-130.

4. Cuppels DA. 1986. Generation and characterization of Tn5 insertion mutations in *Pseudomonas syringae* pv. tomato. Appl Environ Microbiol 51: 323-327.

5. Kovach ME, Elzer PH, Hill DS, Robertson GT, Farris MA, Roop RM, Peterson KM. 1995. Four new derivatives of the broad-host-range cloning vector pBBR1MCS carrying different antibiotic-resistance cassettes. Gene 166:175-176.

6. Pérez-Mendoza D, Aragón IM, Prada-Ramírez HA, Romero-Jiménez L, Ramos C, Gallegos MT, Sanjuán J. 2014. Responses to elevated c-di-GMP levels in mutualistic and pathogenic plant-interacting bacteria. PLoS ONE 9:e91645.

7. Rybtke MT, Borlee BR, Murakami K, Irie Y, Hentzer M, Nielsen TE, Givskov M, Parsek MR, Tolker-Nielsen T. 2012. Fluorescence-based reporter for gauging cyclic di-GMP levels in *Pseudomonas aeruginosa*. Appl Environ Microbiol 78:5060-5069.
