# Supplementary material for: Chemoperception of Specific Amino Acids Controls Phytopathogenicity in Pseudomonas syringae pv. tomato
Source: mBio. 2019 Oct 1;10(5):e01868-19. doi: 10.1128/mBio.01868-19 (PMC6775455; doi:10.1128/mBio.01868-19)
Supplement: TABLE S2 [file mBio.01868-19-st002.docx]

**Table S2.** Primers used

| Primer name | Sequence |  |
| --- | --- | --- |
| 2480LBDFw | 5´-CACCGACTATCGACAACGTCAG-3´ |  |
| 2480LBDRv | 5´-TTAGGCCGAGGTGCGGAACTCG-3´ |  |
| 2480XmaIFw | 5´-AACCCGGGGTATTTATTGTCATC-3´ |  |
| 2480XmaIRv | 5´-TTCCCGGGGAGTTGATGATTTTG-3´ |  |
| 1982XmaIFw | 5’-AACCCGGGATGCAATTTTTCGCGGTT-3’ |  |
| 1982XmaIRv | 5’-TTCCCGGGCAGGTTTTCGAATTCGT-3’ |  |
| 2480CompFw | 5´-AAGAATTCGGGTCTCACGTGTCGAAAA-3´ |  |
| 2480CompRv | 5´-TTGAATTCTTCTGGATGCTCTGCGTC-3´ |  |
|  |  |  |
|  |  |  |
